# Identification of hnRNP-A1 as a pharmacodynamic biomarker of type I PRMT inhibition in blood and tumor tissues

**DOI:** 10.1038/s41598-020-78800-6

**Published:** 2020-12-17

**Authors:** Paul B. Noto, Timothy W. Sikorski, Francesca Zappacosta, Craig D. Wagner, Rocio Montes de Oca, Matthew E. Szapacs, Roland S. Annan, Yan Liu, Charles F. McHugh, Helai P. Mohammad, Steven P. Piccoli, Caretha L. Creasy

**Affiliations:** 1 Experimental Medicine Unit, Oncology R&D, GSK, Collegeville, USA; 2 Protein Mass Spectrometry, In Vitro/In Vivo Translation, Research, GSK, Collegeville, USA; 3 Discovery Analytical, Medicinal Science and Technology, Research, GSK, Collegeville, USA; 4 Epigenetics, Oncology R&D, GSK, Collegeville, USA; 5 Discovery DMPK, In Vitro / In Vivo Translation, Research, GSK, Collegeville, USA; 6 Clinical Biomarkers and Diagnostics, Sun Pharmaceutical Advanced Research Center (SPARC), Princeton, USA

**Keywords:** Cancer, Tumour biomarkers

## Abstract

Arginine methylation has been recognized as a post-translational modification with pleiotropic effects that span from regulation of transcription to metabolic processes that contribute to aberrant cell proliferation and tumorigenesis. This has brought significant attention to the development of therapeutic strategies aimed at blocking the activity of protein arginine methyltransferases (PRMTs), which catalyze the formation of various methylated arginine products on a wide variety of cellular substrates. GSK3368715 is a small molecule inhibitor of type I PRMTs currently in clinical development. Here, we evaluate the effect of type I PRMT inhibition on arginine methylation in normal human peripheral blood mononuclear cells and utilize a broad proteomic approach to identify type I PRMT substrates. This work identified heterogenous nuclear ribonucleoprotein A1 (hnRNP-A1) as a pharmacodynamic biomarker of type I PRMT inhibition. Utilizing targeted mass spectrometry (MS), methods were developed to detect and quantitate changes in methylation of specific arginine residues on hnRNP-A1. This resulted in the development and validation of novel MS and immune assays useful for the assessment of GSK3368715 induced pharmacodynamic effects in blood and tumors that can be applied to GSK3368715 clinical trials.

## Introduction

**Figure 1:**
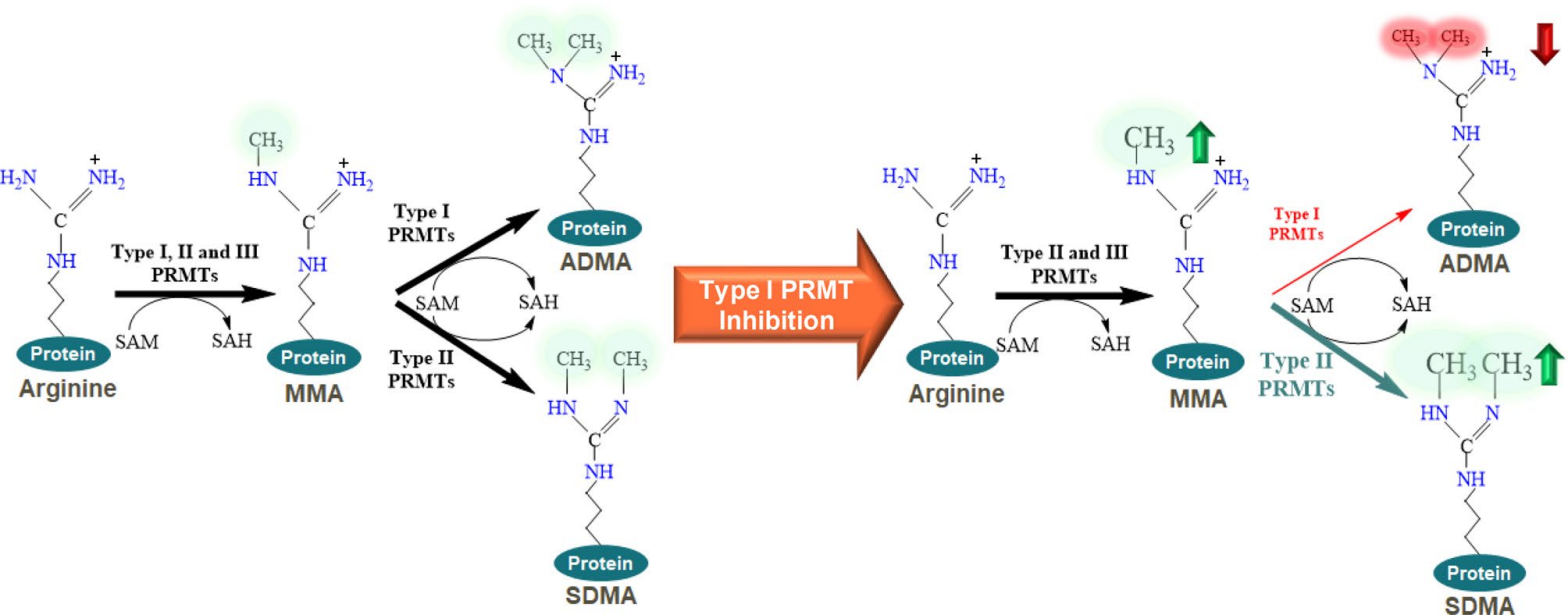
Pharmacology of arginine methylation with type I PRMT inhibition. Schematic of arginine methylation mediated by PRMTs and mechanism of type I PRMT inhibition. The left side of the figure shows normal arginine methylation and the right side illustrates the decrease in ADMA with a concomitant increase in MMA and SDMA upon type I PRMT inhibition.

Arginine methylation is a post-translational modification (PTM) that occurs on a wide range of nuclear and cytosolic proteins spanning histones, signaling molecules and RNA splicing factors^1^. Protein arginine methyltransferases (PRMTs) are a family of enzymes that methylate protein arginine residues in three forms: ω-N^G^-monomethyl-arginine (MMA), ω-N^G^,N^G^-asymmetric dimethyl arginine (ADMA), or ω-N^G^,N’^G^-symmetric dimethyl arginine (SDMA). PRMTs utilize S-adenosyl-L-methionine (SAM) for the transfer of the methyl group to the protein arginine side chain generating S-adenosyl-homocysteine (SAH) and methylated arginine as products (Fig. 1). The PRMT family comprises 10 members that are categorized into four sub-types (type I–IV) according to the type of methylated product from the enzymatic reaction^2^. While type IV enzymes methylate the internal guanidino nitrogen, this activity has only been shown in yeast^3^. Types I–III PRMTs generate MMA as an intermediate product prior to asymmetric or symmetric dimethylation catalyzed by type I and type II PRMTs, respectively. Type I enzymes include PRMT1, PRMT3, PRMT4, PRMT6, which are ubiquitously expressed^4^, and PRMT8 which is primarily restricted to the brain^5^. PRMT1 is the predominant enzyme that catalyzes ADMA formation. Type II PRMTs include PRMT5, responsible for the majority of symmetric dimethylation, and PRMT9. PRMT7 is the only Type III PRMT enzyme known to date and mediates the formation of MMA on substrates that remain monomethylated^6^.

Complete knockout of *Prmt1*^7^ or pharmacological inhibition of type I PRMTs leads^8^ to a decrease in the global levels of ADMA and a concomitant increase of MMA and SDMA due to the compensatory activity of type II PRMTs on shared protein substrates (Fig. 1). Among these, RNA binding proteins (RBPs) involved in RNA processing, splicing, metabolism and transport represent the prominent cellular targets of PRMTs^9–12^. PRMT substrates are typically methylated on arginine residues within glycine-arginine-rich (GAR) motifs, and RBPs, such as small and heterogenous nuclear ribonucleoproteins (sn/hnRNPs), have been extensively characterized accounting for over 60% of ADMA levels in the nucleus (10). Inhibition of type I PRMTs has been shown to yield anti-proliferative effects in multiple tumor types (1, 8). GSK3368715 is an orally bioavailable, SAM uncompetitive, small molecule inhibitor of type I PRMTs (8) that is currently being investigated in a first time in human (FTIH) clinical trial to assess its safety, pharmacokinetics, pharmacodynamics and clinical activity in participants with solid tumors and diffused large B-cell lymphoma (DLBCL) (NCT03666988). While the pharmacodynamic effects of GSK3368715 on global arginine methylation are broad and consistent across cell types, the dynamic range and magnitude of these changes are limited, posing a challenge for use in the clinic. In this study, we describe the identification of hnRNP-A1 as a pharmacodynamic biomarker of type I PRMT inhibition, and the development of novel methodologies to accurately and precisely quantitate changes in the levels of ADMA on hnRNP-A1 in both blood and tumor compartments. These methodologies can be readily implemented in the clinical setting to evaluate GSK3368715 target engagement.

## Results

### Pharmacology of type I PRMT inhibition

**Figure 2:**
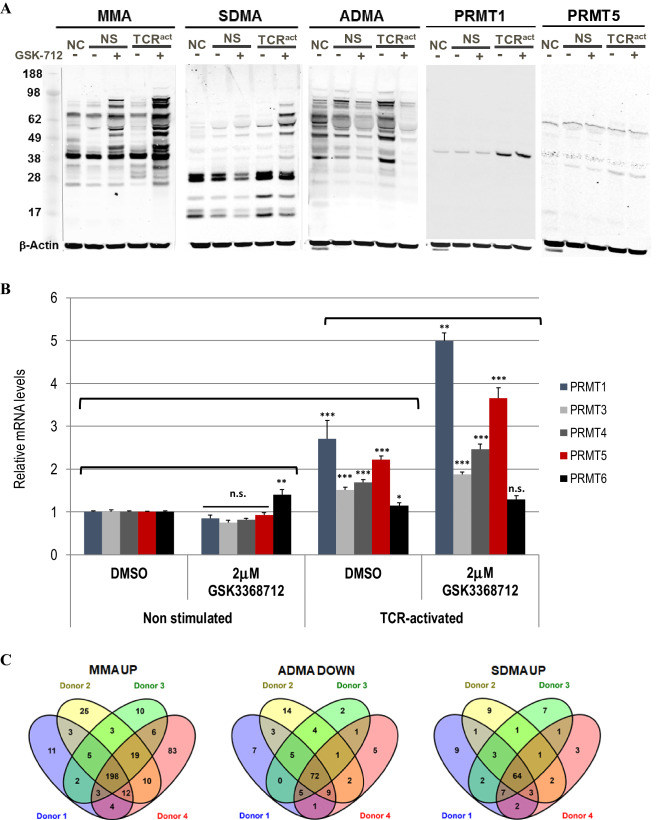

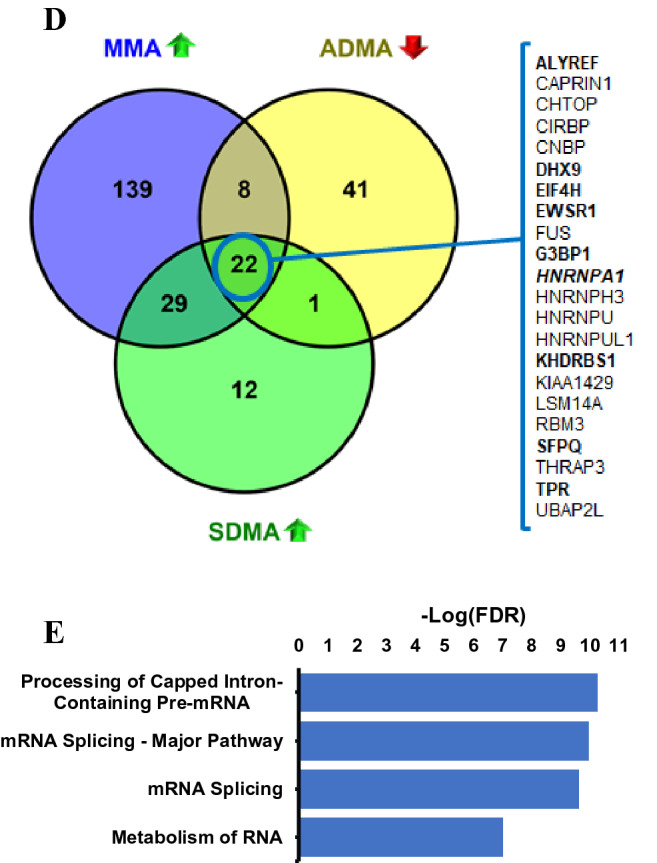
Effects of type I PRMT inhibition in human PBMCs. Western Blot analysis of monomethyl-arginine (MMA), symmetric dimethyl arginine (SDMA), asymmetric dimethyl arginine (ADMA), PRMT1 and PRMT5 (NC, non-cultured, NS, non-stimulated, TCR^act^, T Cell Receptor activated) (**A**) and RT-PCR for several PRMTs (**B**) in non-stimulated and TCR-activated human PBMCs from healthy donors treated with either DMSO or 2 µM GSK3368712 for 72 h (two-tailed Student’s *t* test: *p < 0.05, **p < 0.01, ***p < 0.001; n.s., not significant). (**C–E**) MethylScan analysis of arginine methylation in human PBMCs treated with GSK3368712. (**C**) Venn diagram representation of proteins modulated by the type I PRMT inhibitor at MMA, ADMA and SDMA sites across four healthy donors. (**D**) Common set of proteins where a decrease in ADMA and concomitant increase in MMA and SDMA was observed in all 4 donors. (**E**) Pathway analysis of all proteins that underwent any change (> 2.5 fold) in arginine methylation upon treatment with GSK3368712, compared to DMSO. FDR, False Discovery Rate.

To explore changes to arginine methylation in proliferating and resting human PBMCs, cells cultured with and without T Cell Receptor (TCR)-activation, respectively, were treated for 72 h with GSK3368712, a type PRMT I inhibitor with similar chemical properties to GSK3368715 (8). Treatment led to changes in the levels of global MMA, SDMA and ADMA (Fig. 2A). While a decrease in ADMA and an increase in MMA was observed in non-stimulated PBMCs, upon TCR-activation, treatment with GSK3368712 resulted in a robust decrease in ADMA and an increase in both MMA and SDMA. Evaluation of type I PRMT and PRMT5 gene expression demonstrated increased expression of PRMT5 and the type I PRMTs (PRMT1, PRMT3, PRMT4 and PRMT6) in stimulated PBMCs (Fig. 2B). PRMT1 protein, but not PRMT5 protein increased upon TCR activation [PRMT3 and PRMT4 protein levels were inconclusive (data not shown)], suggesting that increased levels MMA and SDMA with GSK3368712 treatment may be, in part, a consequence of increased type I PRMT levels (Fig. 2A,B).

### Identification of type I PRMT substrates

To overcome technical limitations associated with quantitating global arginine methylation changes upon type I PRMT inhibition, methylated arginine enrichment proteomics (MethylScan)^15^ was used to identify specific proteins that undergo changes in MMA, ADMA and SDMA upon GSK3368712 treatment. Human PBMCs obtained from four healthy donors were treated individually for 72 h with GSK3368712 in the presence of a TCR-activating agent. The purpose of T cell activation was twofold: (a) augmentation of the arginine methylation machinery to maximize identification of sites methylated by type I PRMTs and (b) generate, via clonal expansion, a high number of cells to yield sufficient total protein for enrichment of arginine methylated proteins. Total protein was isolated from the cultured PBMCs, digested with trypsin and immunoprecipitated with antibodies specific to each arginine methylation mark. Immunopurified peptides were resolved and sequenced by liquid chromatography-tandem mass spectra (LC–MS/MS), and fold-changes were calculated relative to DMSO-treated PBMCs. Applying a 2.5-fold change as a cutoff, a decrease of ADMA on 72 proteins common to all four donors was identified (Fig. 2C, Data file 1). In a similar fashion, GSK3368712 treatment resulted in an increase of MMA and SDMA on 198 and 64 proteins, respectively. Interestingly, of the 334 unique proteins that underwent any change in arginine methylation, 22 were identified as bearing a decrease in ADMA with a concomitant increase in MMA and SDMA at one or multiple arginine sites (Fig. 2D), and the majority of arginine methylation changes were detected on proteins involved in mRNA splicing, and RNA processing and metabolism (Fig. 2E). These findings were consistent with results observed in cancer cell lines (8) and suggest that many substrates are conserved across normal PBMCs and cancer.

### Characterization of hnRNP-A1 as a pharmacodynamic biomarker of type I PRMT inhibition

**Figure 3:**
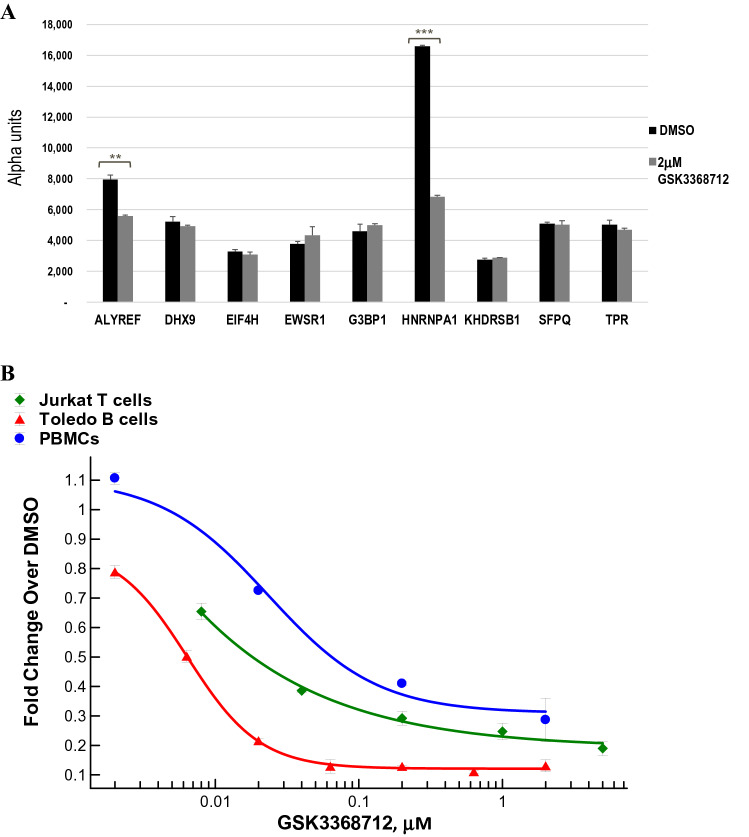
Reduction of ADMA on hnRNP-A1 across normal PBMCs and cancer cells. (**A**) AlphaLISA for candidate substrates identified in the MethylScan study using lysates from TCR-activated human PBMCs treated with either DMSO or 2 µM GSK3368712 for 72 h (two-tailed Student’s *t* test: **p < 0.01, ***p < 0.001; p-values not indicated if no statistically-significant difference was observed). (**B**) ADMA-hnRNP-A1 levels in lysates from non stimulated PBMCs and cancer cell lines, Jurkat and Toledo, treated with increasing concentrations of GSK3368712 for 72 and 48 h, respectively. Data are expressed as the mean ± standard deviation (SD).

To confirm the primary findings from the MethylScan study in TCR-activated PBMCs, we focused on the 22 proteins that underwent decreases in ADMA and increases in MMA and SDMA upon GSK3368712 treatment, consistent with the pharmacology of type I PRMT inhibition. Considering expression of each protein across tissues, access to commercially-available mouse monoclonal antibodies and relative abundance of tryptic peptides identified by mass spectrometry (MS), 9 proteins were selected for further evaluation (ALYREF, DHX9, EIF4H, EWSR1, G3BP1, HNRNPA1, KHDRSB1, SFPQ and TPR; Fig. 2D). Using the same human PBMC lysates analyzed by MethylScan and custom sandwich immunoassays (AlphaLISA) that allow indirect detection of ADMA on each target protein, only a reduction in ADMA on ALYREF and hnRNP-A1 could be confirmed (Fig. 3A). Given the robust signal detected with hnRNP-A1 compared to ALYREF, hnRNP-A1 was selected for further characterization. A number of factors, including, but not limited to, antibody quality or low protein expression may have contributed to the inability to confirm changes in the other potential substrates. HnRNP-A1 is a protein that is involved in several stages of RNA metabolism, from nascent mRNA assembly to RNA processing and splicing^13^, and has been previously described as a substrate of PRMT1^14^. These results were confirmed by analyzing the levels of ADMA-hnRNP-A1 in non-stimulated human PBMCs and two cancer cell lines treated with GSK3368712 for 72 and 48 h, respectively (Fig. 3B).

### Arginine methylation analysis of hnRNP-A1 by mass spectrometry

**Figure 4:**
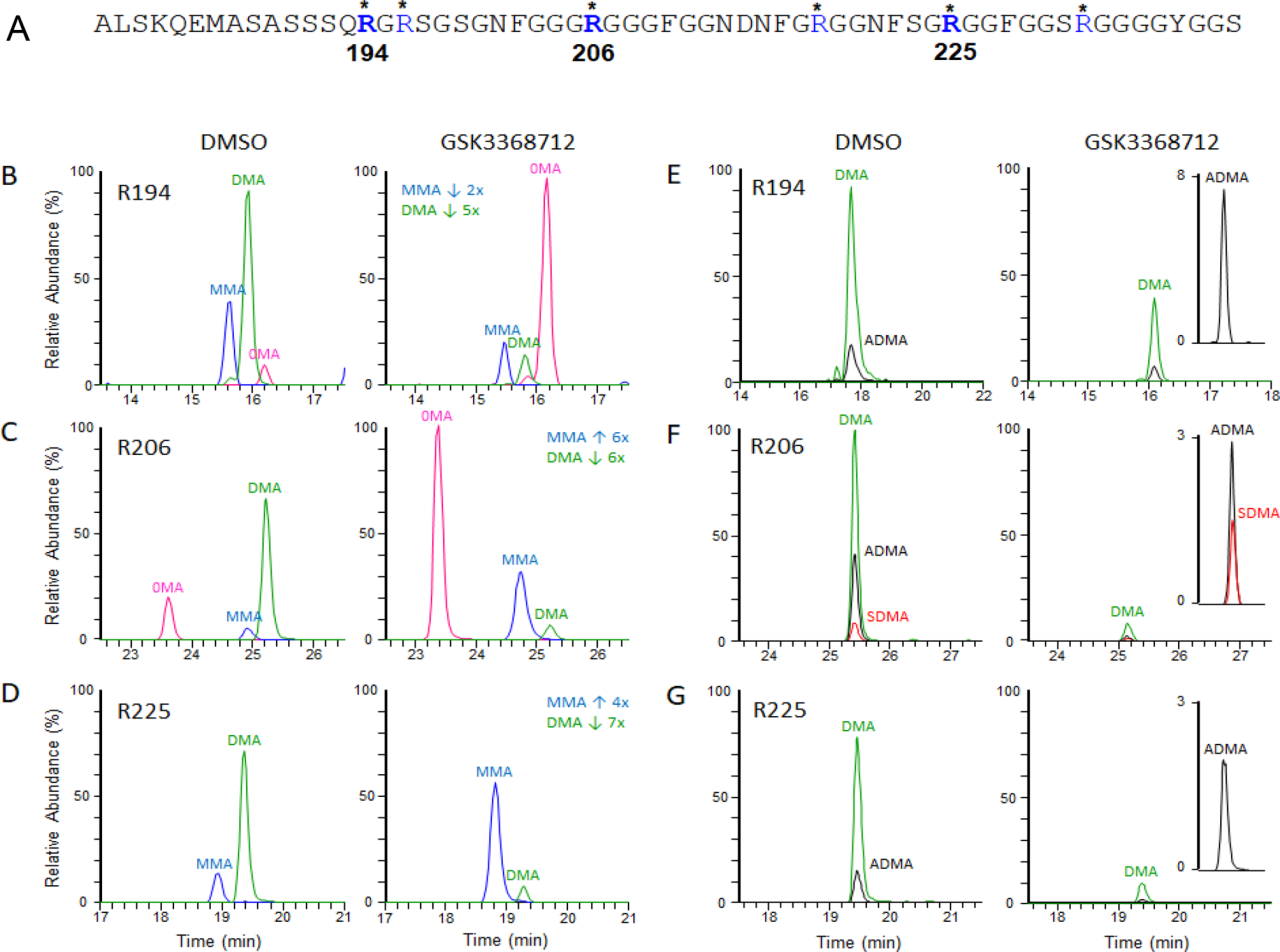
Mass Spectrometry analysis of hnRNP-A1 immunoprecipitated from Toledo cells treated with GSK3368712. **(A)** Amino acid sequence 180–240 of human hnRNP-A1 (* methylated arginine residues identified by MS). (**B, C** and** D**) Chromatographic representation of the methylation forms found at arginine residues 194, 206 and R225 and their relative changes induced by treatment with the type I PRMT inhibitor GSK3368712 (2 µM) for 48 h. 0MA, unmethylated arginine. MMA, monomethylated arginine. DMA, dimethylated arginine. (**E, F** and** G**) Relative levels of asymmetric dimethylarginine (ADMA) and symmetric dimethylarginine (SDMA) with respect to total dimethylarginine 194, 206 and 225 based on generation of diagnostic neutral loss fragment ions.

The results obtained from the MethylScan and AlphaLISA studies from the in vitro culture of human cancer cells and healthy human PBMCs suggested that hnRNP-A1 could be utilized as a clinical biomarker of type I PRMT activity in human blood; therefore, modulation of arginine methylation on hnRNP-A1 by the type I PRMT inhibitor was further characterized. The MethylScan data demonstrated that treatment of TCR-activated human PBMCs with GSK3368712 modulated arginine methylation at various sites on hnRNPA1 (Data File 1), specifically in the GAR-region encompassing amino acids 194–232. This region contains six arginine residues all found to be mono- and/or di-methylated to various extents in the MethylScan data. Also, most of the hnRNP-A1 tryptic peptides identified contained multiple methylated arginine residues, precluding quantitation of methylation on specific residues and making the quantitative interpretation of the data challenging. To overcome this, we carried out an in-depth MS analysis of immunoprecipitated hnRNP-A1 from control and GSK3368712 treated Toledo cells (Figure S1). We identified 12 distinct hnRNP-A1 methylated arginines, six of which are contained in the RG-rich region (R194, R196, R206, R218, R225 and R232; Fig. 4). Based on precursor ion intensity and the quality of the MS/MS spectra, we chose to focus on dimethyl arginine (DMA) sites R194, R206, and R225 found in peptides hnRNP-A1_180-196, hnRNP-A1_197-218 and hnRNP-A1_219-232, respectively. For all three sites, a reduction in DMA following treatment with GSK3368712 was observed (Fig. 4B–D). An increase in the unmethylated forms of R194 and R206 was observed. While a modest decrease in the monomethylated form of R194 was noted, treatment with GSK3368712 led to an increase in monomethylated R206 and R225. To determine whether dimethylation at specific sites was symmetric, asymmetric or a combination, we monitored the generation of diagnostic neutral loss fragment ions specific for either ADMA or SDMA^16^ using parallel reaction monitoring (PRM) LC–MS/MS. Monitoring of the diagnostic neutral loss fragment ions indicate R194 and R225 to be exclusively asymmetrically dimethylated suggesting these residues are solely type I PRMT substrates, while R206 was observed as a combination of ADMA and SDMA, suggesting methylation at this residue is the effect of both type I and type II PRMT activity in these cells (Fig. 4E–G).

Additionally, it was important to investigate the effect of TCR activation on methylation of R225 on hnRNP-A1 in PBMCs. This residue is nearly 100% asymmetrically dimethylated in both non- stimulated and TCR-activated PBMCs with no detectable symmetrical dimethylation (Figure S2). These findings demonstrate that TCR activation does not alter methylation of R225 on hnRNP-A1 in human PBMCs and supports further evaluation of ADM-R225-hnRNPA1 as a pharmacodynamic biomarker of type I PRMT inhibition.

### Development of LC–MS/MS for hnRNP-A1 detection and quantitation in PBMCs from human blood

**Figure 5:**
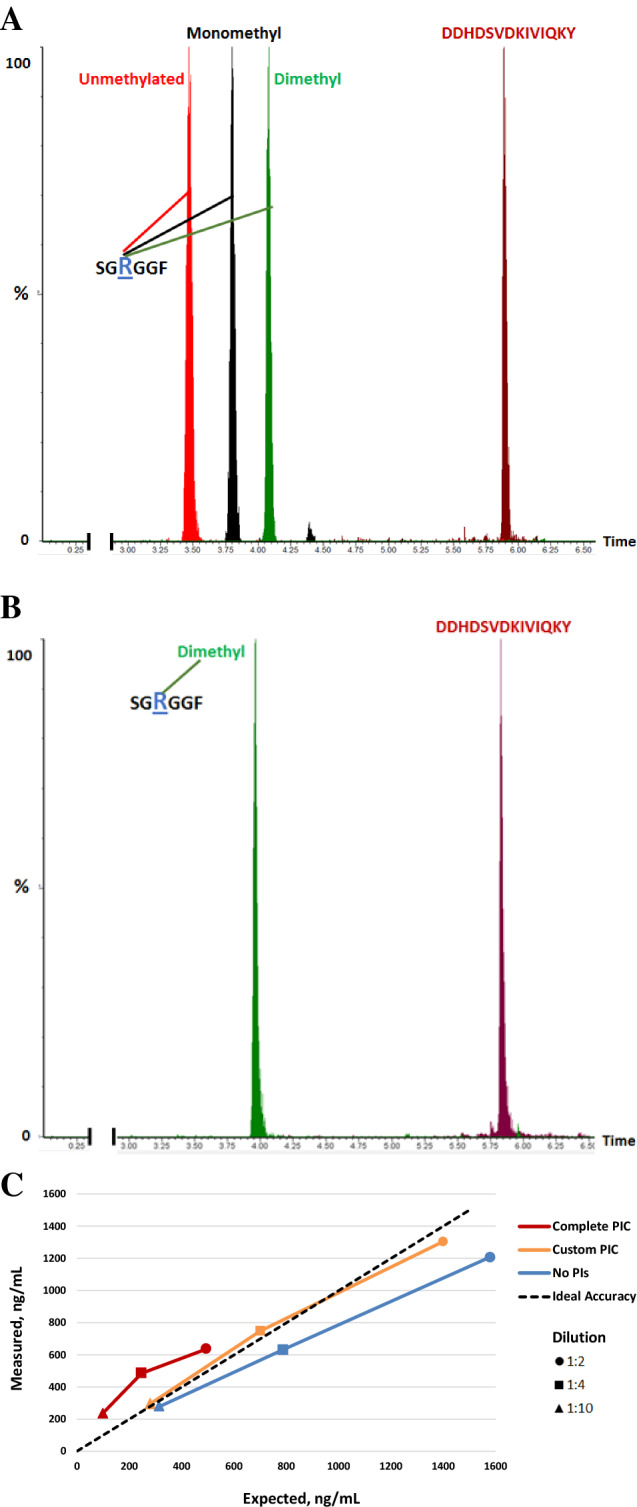
Development of LC–MS/MS method for the detection of hnRNP-A1 in human blood. (**A**) Chromatographic separation of a mixture of synthetic peptides corresponding to hnRNP-A1_223-228_ bearing unmethylated, mono-methylated, asymmetrically-dimethylated arginine 225, and the unmodified hnRNP-A1_154-167_ peptide. (**B**) Detection of chymotryptically-derived hnRNP-A1 peptides in human PBMC lysates, in which hnRNP-R225 is detected in its dimethylated form. (**C**) Determination of method accuracy for the detection of the DM-R225-hnRNP-A1 peptide employing a complete protease inhibitor cocktail (PIC red horizontal line), a PIC without serine protease inhibitors (yellow horizontal line) or no protease inhibitors (blue horizontal line**)** on chymotryptically-digested human PBMC lysates diluted 1:2 (filled circle), 1:4 (filled square) and 1:10 (filled triangle) with PBS. The levels of DM-R225-hnRNP-A1 in undiluted lysates were 988 ng/mL, 3153 ng/mL and 2800 ng/mL in presence of complete PIC, custom PIC and no PIC, respectively.

The lack of R225 in its SDMA form in both PBMCs and cancer cells, and the robust decrease in ADM-R225 observed with GSK3368712 treatment, together with the desire to select a site that would be amenable to both MS and immune-based assay development (see below for details), prompted the selection of R225 for further assessment. A targeted peptide mass spectrometry approach was chosen as it could offer rapid throughput and adequate sensitivity with complete specificity for the dimethylated form of R225 on hnRNP-A1 in human PBMCs. To this end, we developed a novel LC–MS/MS method for the determination of dimethyl (DM)-R225 in human PBMCs and mouse leukocytes protein lysates using the chymotrypsin-derived peptide ‘SG-[R(Me)2]-GGF’ spanning amino acids 223–228. Additionally, to normalize dimethyl-R225 levels to total hnRNP-A1 protein, this method included the quantification of another chymotryptically derived peptide spanning amino acids 154–167 (DDHDSVDKIVIQKY) of human hnRNP-A1 that lacked potential arginine methylation sites. The endoproteinase chymotrypsin, which cleaves at aromatic amino acids, was chosen as the digestion enzyme, instead of the more commonly used enzyme trypsin, which cleaves at arginine and lysine residues. The digestion efficiency of trypsin decreases at arginine sites that are dimethylated^17^, posing a significant challenge for the accurate quantitation of changes in methylated arginine forms. LC–MS/MS analysis of a mixture of synthetic peptides corresponding to hnRNP-A1_223-228_ bearing unmethylated, mono-methylated, or asymmetrically-dimethylated arginine 225 with the hnRNP-A1_154-167_ peptide showed that these modified forms could be distinguished both by mass and chromatographic retention time (Fig. 5A). While all methylation states of R225 can be discriminated with this method, only the asymmetrically dimethylated form of R225 could be identified in human PBMC samples at appreciable levels (Fig. 5B and S3A). This finding indicates that this residue is a high affinity substrate of type I PRMTs and suggests that methylation occurs in a processive fashion. Additionally, the removal of serine protease inhibitors from the lysis buffer significantly improved the sensitivity of the assay by improving chymotrypsin cleavage efficiency (Fig. 5C). Using 12.5 µg of PBMC derived protein lysate, the lower limit of quantification (LLOQ) for both DM-R225-hnRNP-A1 and hnRNP-A1_154-167_ peptides in this assay was determined to be 50 ng/mL (Figure S4). At all quality control sample concentrations examined, the intra- and inter-run precision expressed as % CV were less than or equal to 20% (25% at the LLOQ) (Table S1). Pre-analytical variables associated with both the processing and lysis of PBMCs and stability of hnRNP-A1 peptides over time were also assessed. As shown in Table S2, timing of lysate generation from collection of PBMC isolation and length of storage at -70ºC for up to 8 months do not significantly affect the levels of both hnRNP-A1 peptides.

### Reduction of DM-R225-hnRNP-A1 in human PBMCs and mouse leukocytes by LC–MS/MS

**Figure 6:**
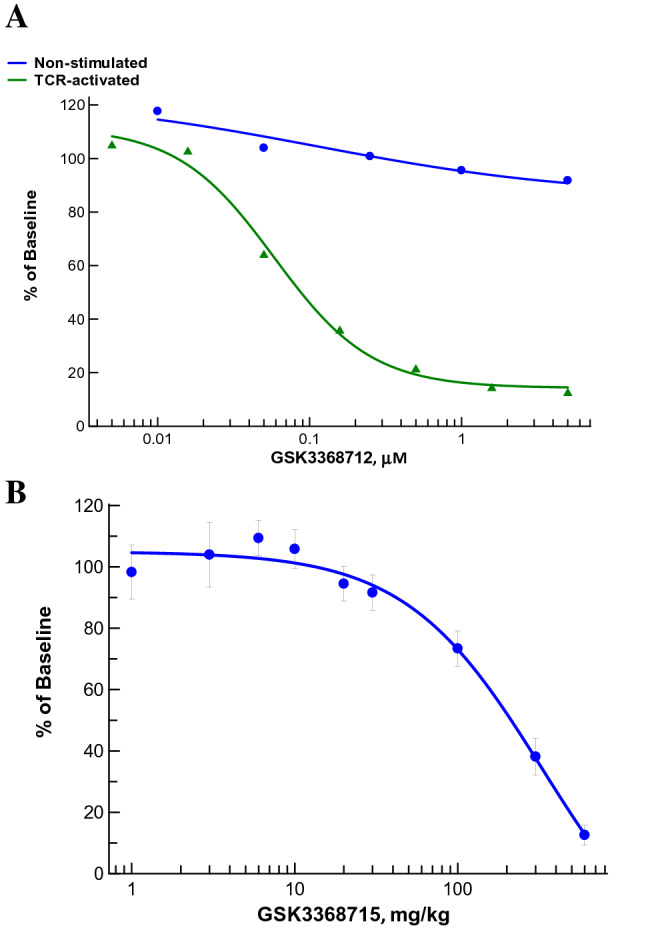
LC–MS/MS quantitation of hnRNP-A1 peptides in human PBMCs and mouse leukocytes. (**A**) Reduction of DM-R225-hnRNP-A1 in human PBMCs treated in vitro with several concentrations of GSK3368712 either without stimulation or in the presence of a TCR-activating agent (CytoSTIM) for 72 h. (**B**) Reduction of DM-R225-hnRNP-A1 in white blood cells from mice (n = 3) treated orally once-daily with several doses of GSK3368715 for 15 days. Levels of DM-R225-hnRNP-A1 were normalized to levels of the hnRNP-A1_154-167_ peptide (total protein) and changes calculated as % of baseline DMSO or vehicle controls. Data are expressed as the mean ± standard deviation (SD).

The optimized LC–MS/MS method was used to assess the levels of DM-R225-hnRNP-A1 in both human PBMCs and mouse leukocytes treated with the type I PRMT inhibitors in vitro and in vivo, respectively. The effects of GSK3368712 were investigated in both non-stimulated and TCR-activated PBMCs. While treatment of non-stimulated human PBMCs led to minimal reduction of DM-R225-hnRNP-A1, a dose-dependent decrease of the biomarker was observed in TCR-activated PBMCs (Fig. 6A). This is consistent with the lack of de novo arginine methylation inhibition in non-proliferating cells, such as non-stimulated PBMCs that have high levels of dimethylated R225-hnRNP-A1. While these findings may appear discordant with the AlphaLISA results shown in Fig. 3B, where treatment with GSK3368712 led to a robust decrease in levels of ADMA-hnRNP-A1 in non-stimulated human PBMCs, it is worth noting that the buffer composition of the AlphaLISA allows protein–protein interactions to be maintained and thus can detect any ADMA on hnRNP-A1 and any of its interacting proteins. Therefore, the decrease in assay signal is directly proportional to any decrease in ADMA on either hnRNP-A1 and/or interacting proteins that bear ADMA residues modulated by type I PRMTs. Given these results, we wanted to demonstrate the utility of the LC–MS/MS method for the assessment of pharmacodynamic changes on DM-R225-hnRNP-A1 in blood from mice treated with the type I PRMT inhibitor. For this in vivo study, and others herein, where quantitative assessment of pharmacodynamic changes are evaluated, the clinical compound, GSK3368715, is used. Treatment with compound once daily for 15 days led to a significant dose-dependent reduction of DM-R225-hnRNP-A1. The highest dose evaluated, 600 mg/kg, yielded an approximate 88% decrease in DM-R225-hnRNP-A1 compared to vehicle-treated animals (Fig. 6B).

### Levels of hnRNP-A1 across blood cell populations

**Figure 7:**
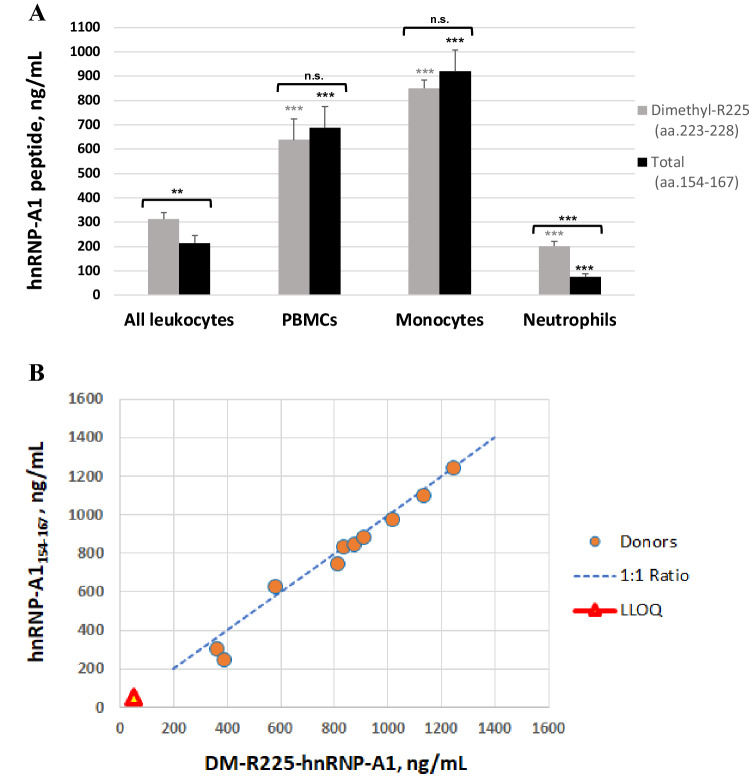
Levels of hnRNP-A1 peptides in human leukocytes. (**A**) Quantitation of DM-R225-hnRNP-A1 and hnRNP-A1_154-167_ peptides across human white blood cell populations from four donors (two males and two females; shown is the average ± S.D.). Levels were determined in all leukocytes (inclusive of granulocytes), PBMCs (comprising lymphocytes, monocytes and natural killer cells), monocytes and neutrophils (two-tailed Student’s *t* test calculated for each peptide in PBMCs, monocytes and neutrophils versus all leukocytes: **p < 0.01, ***p < 0.001, n.s. not significant; brackets indicate a statistically-significant difference between DM-R225-hnRNP-A1 and hnRNP-A1_154-167_ levels). Data are expressed as the mean ± standard deviation (SD). (**B**) Levels of both hnRNP-A1 peptides in human PBMCs obtained from ten donors (five males and five females). LLOQ, lower limit of quantification.

To identify immune cell subtypes with this modification and assess PD changes in DM-R225-hnRNP-A1, we determined the levels of both dimethylated-R225-hnRNP-A1 and hnRNP-A1_154-167_ peptides in monocytes, neutrophils, PBMCs and all white blood cells (WBCs) from four healthy donors using our novel optimized LC–MS/MS method. Upon confirmation of cell purity and relative abundance by flow cytometry analysis (Figure S5 and Table 1), LC–MS/MS analysis of both peptides revealed the highest levels in monocytes and PBMCs, and the lowest levels in neutrophils (Fig. 7A). This finding is consistent with the low levels of hnRNP-A1 detected in all leukocytes, in which granulocytes represent the most abundant cell population (40–70% of all white blood cells;^18^). While this finding was initially hypothesized to be attributable to the high levels of digestive enzymes in neutrophils, addition of neutrophil elastase and collagenase inhibitors failed to improve the detection of hnRNP-A1 (data not shown), suggesting that hnRNP-A1 is likely to be expressed at low levels in neutrophils. This observation is also supported by the work published by Song et al.^19^ showing that the levels of hnRNP-A1 decrease significantly upon granulocytic differentiation. These findings identified PBMCs as the optimal blood compartment to be analyzed for levels of hnRNP-A1 and its PD changes upon treatment with the type I PRMT inhibitor in humans. To evaluate levels of both hnRNP-A1 peptides in human PBMCs, cell preparation tubes (CPT) were used to isolate PBMCs from human blood. As shown in Fig. 7B, the levels of the two peptides in PBMCs obtained from 10 donors range from ~ 300 ng/mL to ~ 1,240 ng/mL. Given that the LLOQ for DM-R225-hnRNP-A1 is 50 ng/mL, this assay allows detection of at least a sixfold-reduction (or ~ 87%) in DMA in cases with the lowest expression of hnRNP-A1.

**Table 1:** Relative levels of leukocytes in blood from four healthy donors.

	% Neutrophils	% B cells	% T cells	% Monocytes
WBCs	41.6 ± 9.3	2.7 ± 1.1	39.3 ± 10.4	3.7 ± 0.9
PBMCs	0.0	3.5 ± 1.7	75.1 ± 3.7	6.5 ± 1.5
Neutrophils	93.6 ± 2.1	0.0	0.1	0.0
Monocytes	0.0	0.0	5.9 ± 10.2	82.7 ± 9.7

WBCs, white blood cells. Neutrophils obtained via negative selection from whole blood and identified as CD45^+^ CD15^+^ CD16^+^. B and T cells identified as CD45^+^ CD3^−^ CD20^+^ and CD45^+^ CD3^+^ CD20^-^, respectively. Monocytes were purified via negative selection from PBMCs and identified as CD45^+^ CD3^−^ CD20^−^ CD14^+^. (average ± S.D. values are shown).

### Generation of a monoclonal antibody specific to ADM-R225-hnRNP-A1

**Figure 8:**
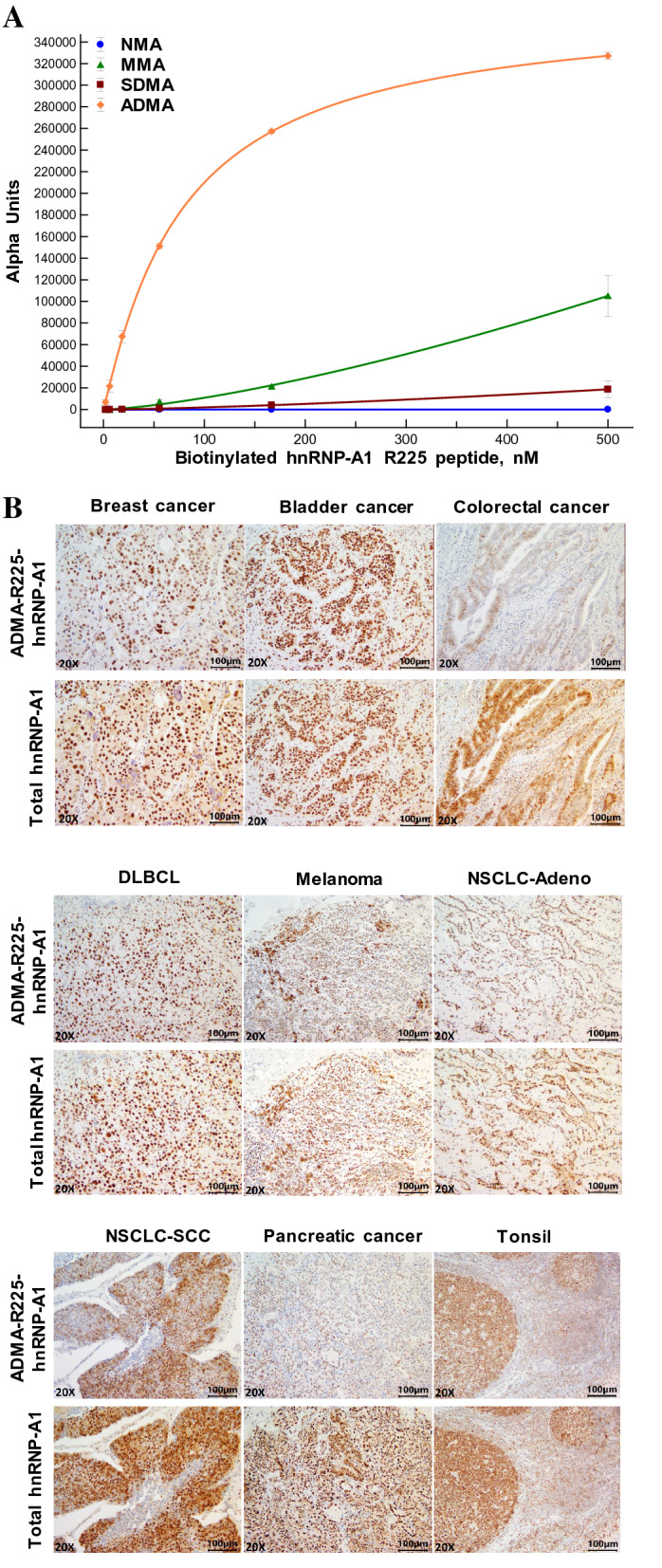

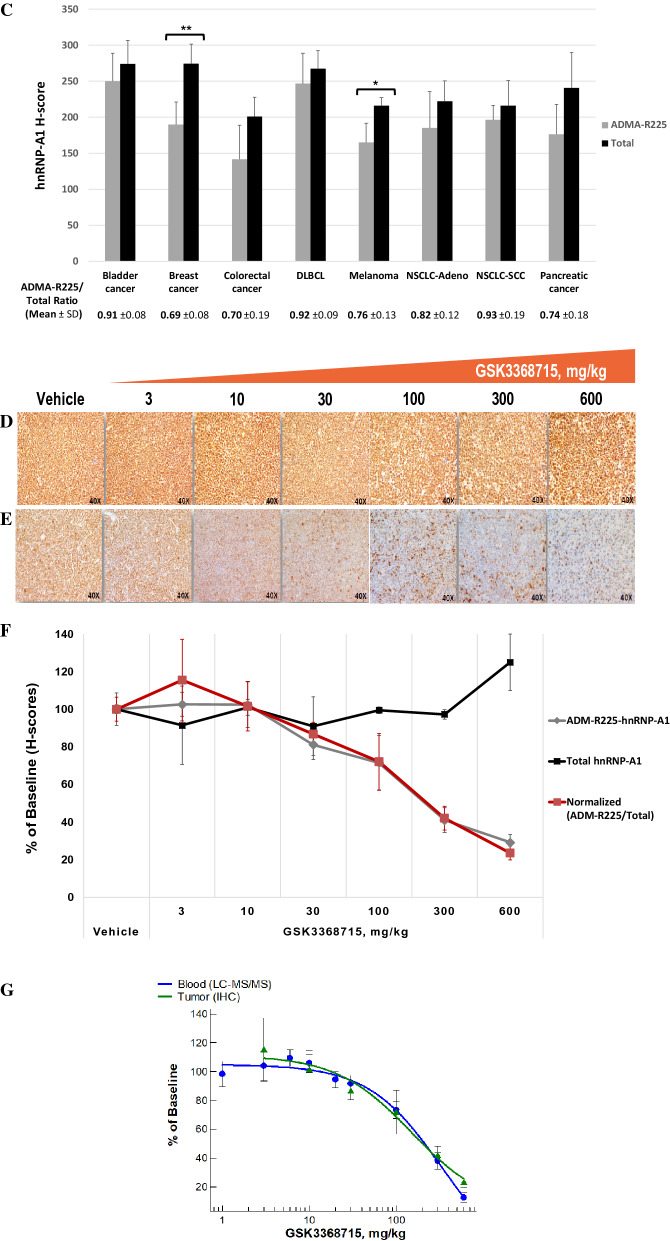
Generation of a monoclonal antibody specific to ADM-R225-hnRNP-A1 and development of IHC assays. **(A)** Characterization of mouse monoclonal antibody (clone 26H3) against all methylated forms of R225-hnRNP-A1 using biotinylated peptides (GNFSG**R**GGFGGSC) in a custom AlphaLISA. NMA, non-methylated arginine. (**B**) Representative images of IHC staining for ADM-R225-hnRNP-A1 and total hnRNP-A1 across a panel of tumor tissues and tonsil. (**C**) Average H-scores for nuclear ADM-R225-hnRNP-A1 and total hnRNP-A1 across eight tumor types (four cases per histology; average ± S.D. are shown) (two-tailed Student’s *t* test: **p < 0.01, ***p < 0.001; *p* values not indicated if no statistically-significant difference was observed); the ADM-R225 to total hnRNP-A1 ratio was obtained from the mean of ratios from all four cases per histology and errors shown as ± S.D (**D, E**) Representative IHC images for total hnRNP-A1 (**D**) and ADM-R225-hnRNP-A1 (**E**) from tumor tissues obtained from mice engrafted with Toledo cells and treated with GSK3368715 orally once-daily for 8 days (n = 3). (**F**) Quantitative analysis of hnRNP-A1 (ADM-R225, total and normalized levels) in Toledo xenografts based on H-score method. Data are expressed as the mean ± standard deviation (SD). (**G**) Changes in levels of ADM-R225-hnRNP-A1 (normalized to total hnRNP-A1) in mouse blood and Toledo xenografts using LC–MS/MS and IHC assays, respectively. DLBCL, Diffuse Large Cell B Cell Lymphoma; NSCLC, Non-Small Cell Lung Cancer; Adeno, Adenocarcinoma; SCC, Squamous Cell Carcinoma.

To assess the potential use of hnRNP-A1 as a clinical biomarker of target engagement in human tissues, we explored the opportunity to develop an antibody that is specific to asymmetrically dimethylated R225 of human hnRNP-A1. The choice of R225 over R194 and R206 was based on several factors, such as, but not limited to, lack of symmetric dimethylation, higher immunogenicity potential and higher likelihood of selectivity for the asymmetrically dimethyl form over non-methylated, mono-methylated and symmetrically dimethylated arginine. A synthetic peptide corresponding to amino acids 220–231 of hnRNP-A1 (GNFSG**R**GGFGGS) bearing asymmetrically dimethylated R225 was used to immunize mice. A custom electrochemiluminescence method (MesoScale Discovery, MSD) was used to determine the selectivity of hybridoma-derived supernatants for the ADMA form of R225 by employing biotinylated peptides bearing all forms of arginine methylation on residue R225, including the non-methylated form as a control. One clone (26H3) was selected from the primary screen (Table S3) based on both affinity and selectivity for the ADM-R225-hnRNP-A1 peptide over non-methylated, MM- and SDM-R225 hnRNP-A1 peptides. Purified antibody from the 26H3 clone was further characterized against several concentrations of peptides bearing all three methylation modifications of R225 in a custom AlphaLISA (Fig. 8A). Clone 26H3 showed complete selectivity for the ADMA over the non-methylated and symmetrically dimethylated forms of R225-hnRNP-A1 while retaining some affinity for the mono-methylated form (K_d_ > 500 nM). By Western blot, 26H3 detects a single band at the expected molecular weight (34–38 Kd) for hnRNP-A1 in PBMCs and Toledo cells (Figure S6). Additionally, the use of recombinant hnRNP-A1 expressed in prokaryotic and eukaryotic cells further demonstrates the specificity of these antibodies for the dimethylated form of the protein as shown by the detection of ADMA on hnRNPA1 in C-myc/DDK-tagged hnRNP-A1 expressed in HEK293 human kidney cells, but not in hnRNPA1 expressed in E. coli where arginine methyltransferases do not exist. The presence of multiple bands in the HEK-expressed material can be attributed to the presence of multiple recombinant hnRNPA1 degradation products. Together, these data demonstrate that 26H3 is highly specific for ADMA-hnRNPA1.

### Development of hnRNP-A1 IHC assays

The preliminary characterization of the novel antibody introduced the opportunity to develop an immunohistochemistry (IHC) method to monitor the pharmacodynamic effects of the type I PRMT inhibitor on hnRNP-A1 in human tumor tissues. Clone 26H3 was tested at several concentrations and conditions in human normal and tumor tissues along with a commercially available antibody for the detection of total hnRNP-A1 for use in normalization (Fig. 8B). The levels of both asymmetrically-dimethylated-R225 and total hnRNP-A1 in the nuclear and cytoplasmic compartments of tumor cells were quantitated using the H-score methodology. IHC results across eight tumor types revealed localization of hnRNP-A1 to the nucleus, with very little detection in the cytoplasm (Data file 2) while mouse and rabbit IgG isotype control antibodies revealed no stain in the histologies examined (Figure S7). The overlapping patterns of expression (Fig. 8B) and comparable levels of stain intensity (Fig. 8C) of ADM-R225- and total hnRNP-A1 across all tumor types suggest that the majority of the hnRNP-A1 protein pool is nearly fully asymmetrically-dimethylated at the R225 site. Only in breast cancer and melanoma did we observe a small, yet statistically-significant, difference in levels of ADMA and total hnRNP-A1, as indicated by the lower ADM-R225/Total ratios, suggesting that hnRNP-A1 may not be completely asymmetrically-dimethylated in these two tumor types. However, additional studies in a larger sample set would be required to determine whether the effect is indeed tumor type-specific or the result of small sample size. To evaluate the pharmacodynamic effects of the type I PRMT inhibitor, optimized IHC conditions for both ADM-R225-hnRNP-A1 and total hnRNP-A1 antibodies were employed on formalin-fixed tumor tissues from a mouse xenograft model using the Toledo human diffuse large B-cell lymphoma (DLBCL) cell line. Treatment of engrafted SCID mice with GSK3368715 administered once daily for 8 days led to a significant reduction of the asymmetric dimethyl mark on R225 of hnRNP-A1 in tumors tissues (Fig. 8D–E). Importantly, the levels of total hnRNP-A1 protein were not affected by compound treatment at doses where tumor regression was observed, except at the highest dose tested (600 mg/kg) (Fig. 8G, Table S4, Figure S8). However, upon normalization of ADMA to total hnRNPA1 levels, a dose proportional decrease in ADMA-hnRNPA1 is still observed at the highest dose (600 mg/kg) (Fig. 8F).

Finally, comparison of the reduction in ADM-R225-hnRNP-A1 in mouse blood (Fig. 6B) to that observed in tumor xenografts demonstrates similar reduction in ADMA-hnRNPA1 in tumor and circulation following multiple days of GSK3368715 treatment using these distinctly different assays (Fig. 8G). While these studies were conducted in different strains of mice, it is possible to compare pharmacodynamic changes across studies in this instance since the exposure of GSK3368715 was quite similar in each strain (Table S5), and ADMA-hnRNPA1 was assessed after many days of dosing where steady state levels of drug would be reached.

## Discussion

Type I PRMTs catalyze the formation of asymmetric dimethyl arginine (ADMA) on a wide range of cellular substrates spanning histones, signaling molecules and RNA splicing factors. Pharmacological inhibition of type I PRMTs has been shown to yield anti-proliferative effects in multiple tumor types (8). The type I PRMT inhibitor GSK3368715 is currently being evaluated in a first time in human clinical trial to investigate the safety, pharmacokinetics, pharmacodynamics and clinical activity in participants with solid tumors and DLBCL (NCT03666988). The complex bimodal pharmacology of type I PRMT inhibition, characterized by a decrease in ADMA with a concomitant increase in MMA and SDMA, posed a challenge for the development of clinical biomarker assays based on the detection of global methylated arginine levels. While Western blots can provide useful qualitative information in a pre-clinical setting, their use for clinical samples is suboptimal due to both procedural complexity and semi-quantitative nature associated with the lack of uniform signal linearity across broad molecular weight ranges. To overcome this challenge, we explored the effect of type I PRMT inhibition in human PBMCs employing a proteomic approach aimed at identifying substrates that could be monitored for the reduction in asymmetric dimethylation at specific arginine sites. Our study identified several candidate substrates as potential PD biomarkers of type I PRMT inhibition. Through custom immunoassays, hnRNP-A1 was identified as a promising biomarker of type I PRMT inhibition. HnRNPs are ubiquitously expressed across tissues with varying levels of abundance^20^, and hnRNP-A1 has been widely investigated for its involvement in all key steps of RNA metabolism^21^ with a focus on how PTMs, such as phosphorylation^22–24^, sumoylation^25^ and methylation^26,27^, modulate its function as a key regulator of gene expression. The reduction of ADMA on hnRNP-A1 was demonstrated in both cancer lines and human PBMCs and this prompted us to develop an LC–MS/MS method aimed at detecting and quantifying dimethylarginine 225 levels on hnRNP-A1 in human PBMCs. The advantage of this method over immunodetection assays is the ability to fully discriminate all forms of arginine methylation on R225, avoiding potential cross-reactivity with MMA which increases upon type I PRMT inhibition. The novelty of this LC–MS/MS method is in the use of chymotrypsin to digest proteins. Typically, trypsin is used; however, trypsin cleaves at arginine residues, and the efficiency of cleavage is diminished at post-translationally modified arginines. Furthermore, removal of serine protease inhibitors from cell lysates resulted in a significant improvement of assay robustness and linearity, making the method more amenable for use in a clinical setting. Using this LC–MS/MS assay, decreases in DMA-R225-hnRNP-A1, but not hnRNP-A1, are observed in both human PBMCs and mouse leukocytes after type I PRMT inhibition (Fig. 6). We observed that PBMCs and monocytes have higher levels of hnRNP-A1 expression and dimethylated R225 than neutrophils and total white blood cells (Fig. 7A). This enabled identification of cell preparation tubes (CPT) as the optimal collection method to enrich for cell populations (PBMCs) expressing hnRNP-A1. Finally, given the LLOQ of the LC–MS/MS assay, the levels of DMA-hnRNP-A1 in PBMCs across donors fall in a range that allows detection of at least an 87% reduction in DMA-R225-hnRNP-A1 even in cases with the lowest levels of the protein. Importantly, the specificity of this assay allowed us to determine that R225-hnRNP-A1 is present in both human PBMCs and Toledo cells in its asymmetrically-dimethylated form only, suggesting that this residue is a high affinity substrate of type I PRMTs. Establishing the utility of hnRNP-A1 as a biomarker of target engagement in surrogate tissue, such as PBMCs, prompted us to investigate the pharmacodynamic effects of type I PRMT inhibition on levels of ADMA-hnRNP-A1 in tumor tissues. Mass spectrometric characterization of hnRNP-A1 in cancer cells allowed identification of R220-R231 as a candidate epitope for the generation of a mouse monoclonal antibody specific to the asymmetric dimethylated form of R225 on hnRNP-A1. We were able to identify an antibody clone, 26H3, with high affinity and selectivity for ADM-R225 over unmethylated-, symmetrically dimethylated- and monomethylated- R225 (Fig. 8A). This antibody was used to develop a novel IHC assay for the detection of ADMA-hnRNP-A1 in tumor tissues (Fig. 8B). Additionally, an IHC assay for total hnRNP-A1 was developed in parallel to normalize for the changes in ADM-R225-hnRNP-A1. Both assays were used to assess the effects of GSK3368715 in Toledo tumor xenografts, and successfully demonstrated the dose-dependent decrease in levels of ADM-R225, but not total, hnRNP-A1 in tumor tissue. Importantly, since LC–MS/MS and IHC methods allowed us to quantitate the pharmacodynamic effect of the type I PRMT inhibitor on hnRNP-A1 in mouse PBMCs and Toledo xenografts, we were able to determine that the dose dependent reduction in ADM-R225-hnRNP-A1 in both blood and tumor tissues is similar in these two compartments following multiple days of dosing (Fig. 8G). In summary, this work led to the development of fit-for-purpose LC–MS/MS and IHC assays as two novel methodologies that allow for quantitation of changes in asymmetrically-dimethylated R225 on human hnRNP-A1 with high specificity and sensitivity in both surrogate and tumor tissues. These assays are currently being used to assess pharmacodynamic changes in patients treated with GSK3368715 (clinical trial NCT03666988).

## Materials and methods

### Culture of human PBMCs and cancer lines

Cryopreserved PBMCs (AllCells, PB005F) were cultured in AIM-V AlbuMAX medium (Thermo Fisher Scientific, #31035025) at 37 °C in 5% CO_2_. GSK3368712 was synthesized and purified as previously described (8) and used at various concentrations either in the absence (non stimulated) or presence of human CytoSTIM (TCR-activated) (Miltenyi, #130-092-172) at a final concentration of 1 µg/mL for 72 h. Jurkat and Toledo cells (ATCC, TIB-152 and CRL-2631, respectively) were cultured in RPMI-1640 (Thermo Fisher Scientific, #11875093) supplemented with 10% Fetal Bovine Serum (FBS, SAFC, #12176C) at 37 °C in 5% CO_2_. The identity of the two cell lines was validated by short tandem repeat (STR) profiling and both were confirmed negative for mycoplasma. GSK3368712 was used at various concentrations for 48 h. Dimethyl sulfoxide (DMSO, Sigma-Aldrich, #34869) was used in all in vitro experiments as a negative pharmacological control at a final concentration of 0.1%. Human biological samples were sourced ethically and their research use was in accord with the terms of the informed consents. The use of human tissue samples was reviewed and approved by GSK Research & Development Compliance (RDC) Human Biological Sample Use Committee.

### Western blot analysis

For whole-cell lysis, harvested PBMC culture pellets were washed with Dulbecco’s phosphate-buffered saline (Thermo Fisher Scientific, #14190144), lysed in CellLytic M buffer (Sigma-Aldrich, #C2978) + 1X Protease Inhibitor Cocktail (Sigma-Aldrich, P8340), and sonicated for 10 min with intervals of 30 s on and 30 s off, and incubated on ice for 10 min. Homogenized samples were applied to a QIAshredder column (Qiagen, #79656), and centrifuged at 12,000 × RCF for 2 min. Protein extracts were quantified using the BCA Protein Assay Kit (Pierce, #23225), and 30–45 µg of protein were resolved by SDS–polyacrylamide gel electrophoresis (PAGE) and blotted onto nitrocellulose membrane (Thermo Fisher Scientific, #IB301001). Blots were blocked in Odyssey Blocking Buffer (Li-Cor, #927-40000) for 60 min at room temperature and incubated with the respective primary antibodies in Odyssey blocking buffer with 0.1% Tween-20 (Sigma-Aldrich, #P1379) overnight at 4 °C with rocking. Blots were washed thoroughly in PBS with 0.2% Tween 20 and incubated with fluorophore–conjugated secondary antibodies in Odyssey blocking buffer with 0.2% Tween-20 at room temperature for 1 h. Blots were washed thoroughly in PBS with 0.2% Tween 20 and scanned using Li-Cor Odyssey imager. The following primary antibodies were used: anti-monomethylated arginine (mme-R, Cell Signaling Technology (CST), #8015, 1:1,000 dilution), anti-symmetric dimethylated arginine (sdme-RG, CST, #13222S, 1:1,000 dilution), and anti-asymmetric dimethylated arginine (clone D10F7A10, CST, 1:1,000 dilution), anti-PRMT1 (clone F339, CST , #2453, 1:1,000 dilution), anti-PRMT5 (CST, #2252, 1:1,000 dilution), anti-hnRNP-A1 (Abcam, #ab177152, 1:1,000 dilution) and anti-ADM-R225-hnRNP-A1 (clone 26H3; custom development for GSK by GenScript; 1 µg/mL) and anti-β-actin (Sigma-Aldrich, #A2228 1:10,000 dilution); the secondary antibodies, IRDye 800CW Goat anti-Rabbit IgG (H + L) and IRDye 680CW Goat anti-Mouse IgG (H + L), were purchased from Li-Cor (#92532211 and #92568020), and used at a dilution of 1:20,000. Recombinant human hnRNP-A1 protein expressed in E.coli (Abcam, #ab123212) and HEK293 cells (Origene, #TP303314) were used as controls. Blots were imaged using Odyssey CLx and processed with the Image Studio Software.

### Quantitative PCR

Non stimulated and TCR-activated human PBMCs were cultured with either 0.1% DMSO or 2 µM GSK3368712 in AIM-V AlbuMAX medium for 72 h and then harvested for isolation of total RNA using the RNeasy Mini Kit, according to the manufacturer’s protocol (Qiagen, #74104). RNA was quantitated using NanoDrop (Thermo Fisher Scientific). All samples were then reverse-transcribed to cDNA and subjected to quantitative PCR using TaqMan RNA-to-CT 1-Step Kit (Thermo Fisher Scientific, #4392938) and triplicate reactions were run on ABI ViiA 7 (Applied Biosystems) according to the manufacture’s protocol. PCR amplifications were performed with specific TaqMan primers for PRMT1, PRMT3, PRMT4, PRMT5 and PRMT6 (Thermo Fisher Scientific, #Hs01587651g1, Hs00411605m1, Hs00406354m1 Hs01047356m1, Hs00250803s1, respectively). mRNA expression of target genes was normalized to the endogenous reference 18S gene (Hs03003631_g1) and the relative fold change from non stimulated, DMSO-treated control PBMCs was calculated using the 2^^ΔΔCT^ method.

### Identification of differentially methylated type I PRMT substrates in human PBMCs

Cryopreserved human PBMCs (AllCells, #PB005F) from 4 healthy donors, 2 females and 2 males, were cultured in AIM-V AlbuMAX medium with either 0.1% DMSO or 2 µM GSK3368712 in the presence human of CytoSTIM at a final concentration of 1 µg/mL for 72 h. Cells were collected, washed twice in DPBS, lysed with a solution containing 20 mM HEPES (pH 8.0), 9.0 M Urea, 1 mM sodium orthovanadate (activated), 2.5 mM sodium pyrophosphate and 1 mM ß-glycerol-phosphate, and flash frozen. Cellular extracts prepared in urea lysis buffer were reduced, alkylated and digested with trypsin. 12 mg total protein for each sample was desalted over SEP PAK C18 columns and split into 3–4 mg aliquots for enrichment with MMA motif [mme-RG] immunoaffinity beads (CST, #12235), a mixture immunoaffinity beads conjugated to ADMA motif [adme-R] and custom ADMA antibody (clone D10F7A10) (CST, #13474; modified method), and SDMA motif [sdme-RG] immunoaffinity beads (CST, #13563). Enriched peptides were loaded directly onto a PicoFrit capillary column packed with Magic C18 AQ reversed-phase resin. Two non-sequential replicate injections were run for each enrichment. Proteomic analysis was carried out using the MethylScan method as previously described^28^. Each enriched sample was analyzed by LC–MS/MS in a data-dependent manner on a Thermo Orbitrap Q Exactive mass spectrometer using a top-twenty MS/MS method with a dynamic repeat count of one, and a repeat duration of 30 s. Peptides were eluted using a 90- or 120-min linear gradient of acetonitrile in 0.125% formic acid delivered at 280 nL/min. Peptide sequences were identified by searching MS/MS spectra against the SwissProt *Homo sapiens* database using SEQUEST^29^ with a mass accuracy of 5 ppm for precursor ions and 0.02 Da for product ions. Enzyme specificity was set to semi-trypsin with up to four mis-cleavages allowed. Cysteine carboamidomethylation was specified as a fixed modification, oxidation of methionine and mono- or di-methylation on arginine residues were allowed as variable modifications. Reverse decoy databases were included for all searches to estimate false discovery rates (FDR), and filtered using a 2.5% FDR. All quantitative results were generated using Skyline^30^ to extract the integrated peak area of the corresponding peptide assignments. Accuracy of quantitative data was ensured by manual review in Skyline or in the ion chromatogram files. Fold changes were calculated for each treatment relative to the DMSO control. % CV values between replicates of each condition were calculated as a measure of analytical reproducibility. For each comparison, we set the fold change observed between two conditions to be at least 2.5 fold and for the methylated peptides identified to be present in all samples from the four donors. Protein lists were merged for all 3 methyl marks and duplicates were removed to obtain a master list of proteins with an increase in the monomethyl- and symmetric dimethyl marks (for MMA and SDMA immunoprecipitations) and a concomitant decrease in the dimethyl mark (for ADMA immunoprecipitations) on the detected peptide (Data File 1). Overlaps were generated using Venny 2.1 (https://bioinfogp.cnb.csic.es/tools/venny/) to obtain a list of changed proteins that were common across PBMCs from all four donors. Analysis of overlap enrichment on the list of gene names of the common proteins was performed using the Reactome biological pathway webtool.

### Immunodetection of ADMA on candidate type I PRMT targets by AlphaScreen

TCR-activated PBMCs employed for the MethylScan study were used to generate lysates as described above for Western blot analysis. Protein concentrations were determined using the BCA Protein Assay Kit. The anti-ADMA antibody (CST, clone D10F7A10) was biotinylated using the EZ-Link Sulfo-NHS-Biotin kit (Thermo Fisher Scientific, #21326) according to the manufacturer’s instructions. 1 µg of lysates, in triplicates, was incubated with the biotinylated anti-ADMA antibody, at a concentration of 0.5 µg/mL, and antibodies specific to ALYREF (Abcam, #ab6141), DHX9 (Abcam, #ab54593), EIF4H (Abcam, #ab77455), EWSR1 (Abcam, #ab54708), G3BP1 (Abcam, #ab56574), HNRNPA1 (Abcam, #ab5832), KHDRSB1 (Abcam, #ab56836), SFPQ (Abcam, #ab11825) and TPR (Abcam, #ab58344) at a concentration of 1 µg/mL. After a 45-min incubation at room temperature, streptavidin-coated acceptor beads (Perkin Elmer, #AL125C) and anti-mouse IgG-coated donor beads (Perkin Elmer, #AS104) were added to respective wells of 96 well microplates (Corning, #3693), at a final concentration of 20 µg/mL and incubated for an additional 45 min at room temperature. All solutions were prepared in AlphaLISA universal buffer (Perkin Elmer, #AL001F) and microplates were shaken after each addition and centrifuged prior to luminescence detection on an Envision microplate reader (Perkin Elmer).

### Quantitation of ADMA on hnRNP-A1 in human cells by AlphaLISA

Lysates from Toledo and Jurkat cancer cells and PBMCs treated with GSK3368712 were generated as described above for Western blot analysis and protein concentrations were assessed using the BCA Protein Assay Kit. 1 µg of lysates, in triplicates, was incubated with a custom anti-ADMA antibody (clone D10F7A10; provided to GSK by CST as a beta product) and an antibody specific to hnRNP-A1 (Abcam, #ab5832), at a final concentration of 1 µg/mL in AlphaLISA universal buffer for 1 h at room temperature. Anti-rabbit-IgG-coated acceptor beads (Perkin Elmer, #AL104) were added at a final concentration of 20 µg/mL and incubated for 1 h at room temperature. Anti-mouse IgG donor-coated beads (Perkin Elmer, #AS104) were added at a final concentration of 40 µg/mL and incubated for an additional 2 h at room temperature. Microplates were shaken after each addition and centrifuged prior to luminescence detection on an Envision microplate reader.

### Analysis of hnRNP-A1 arginine methylation in Toledo cells by LC–MS/MS

Toledo cells were cultured with 0.1% DMSO or 1 μM GSK3368712 for 48 h, collected and lysed in CellLytic M buffer. hnRNP-A1 was immunoprecipitated with mouse anti-hnRNP-A1 antibody (Abcam, #ab5832) using the Pierce Classic Magnetic IP/Co-IP Kit (Thermo Fisher Scientific, #88804) per manufacturer’s instructions. Immunoprecipitated eluates were precipitated with acetone, separated by SDS-PAGE and visualized by Coomassie staining. The hnRNP-A1 bands were excised, reduced using TCEP (Thermo, #77720), alkylated with iodoacetamide (Sigma, #I1149) and digested overnight with trypsin at 37 °C (Promega, #V5111). The following method was performed as previously described (8). After organic extraction, samples were injected on an Easy nLC1000 UHPLC system (Thermo Scientific). The nanoLC was interfaced to a Q- Exactive Hybrid Quadrupole-Orbitrap Mass Spectrometer (Thermo Scientific). Tryptic peptides were separated on a 25 cm × 75 μm ID, PepMap C18, 3 μm particle column (Thermo Scientific) using a 40 min gradient of 2–30% acetonitrile/0.2% formic acid and a flow of 300 nL/min. MS-based peptide sequencing was accomplished by tandem mass spectrometry using data dependent LC–MS/MS. Uninterpreted tandem MS spectra were searched for peptide matches against the human UniProt protein sequence database using Mascot (Matrix Science). Carboamidomethylation was selected as a fixed modification on cysteine residues. Oxidation on methionine and methylation and dimethylation on arginine residues were selected as variable modifications. MS/MS spectra for methylated peptides were manually validated to confirm the site of mono or dimethylation. Integrated peak areas from Extracted Ion Chromatograms (XICs) from the MS scan were used to monitor un-, mono- and dimethylation in control and inhibitor treated samples. Identified hnRNP-A1 methylation sites were further interrogated using a parallel reaction monitoring (PRM) method targeting the dimethylated peptides. Diagnostic ions for either ADMA (neutral loss of 45.0578) or SDMA (neutral loss of 31.0422) were monitored and allowed the determination of the ADMA/SDMA status.

### Development of LC–MS/MS method for quantitation of DMA-R225-hnRNP-A1 in PBMCs

A targeted LC–MS/MS method was developed for the detection of unmethylated, monomethylated, and dimethylated Arginine 225 on hnRNPA1 in human PBMC lysate using chymotryptically derived peptides ‘SGRGGF’, ‘SG-[R(Me)]-GGF’, and ‘SG-[R(Me)2]-GGF’ spanning amino acids 223–228, as well as for the determination of total hnRNPA1 in human PBMC lysate using a chymotryptically derived peptide ‘DDHDSVDKIVIQKY’ spanning amino acids 154–167. Briefly, 12.5 µg of human PBMC protein lysate in RIPA buffer were spiked with synthetic, stable isotope labeled standard peptides corresponding to SG-[aR(Me)2]-GGF and DDHDSVDKIVIQKY, and digested overnight at 24 °C with 10 µg of the endoproteinase enzyme chymotrypsin in digestion buffer (100 mM Tris HCl, pH 8.0, 10 mM CaCl_2_, 20% Acetonitrile). The samples were then acidified, and peptides were enriched using strong cation mixed mode solid phase extraction. The chymotryptically derived peptides were chromatographically separated on a 2.1 mm × 100 mm Peptide BEH column (Waters) using a gradient of 0–11% acetonitrile/0.1% formic acid in 4.5 min, then 11–30% acetonitrile/0.1% formic acid in 2 min at a flow rate of 600 µL/min, and analyzed with a Waters Xevo-TQS mass spectrometer and a multiple reaction monitoring method (Table S6). Calibration standards to establish the quantitative range of the method for SG-[R(Me)2]-GGF and DDHDSVDKIVIQKY were prepared with hnRNPA1 reference protein (Origene Cat#TP303314). UPLC-MS/MS data were acquired and processed (integrated) using the software application Masslynx (Version 4.1, Waters Corporation). Calibration plots of analyte/internal standard peak area ratio versus SG-[R(Me)2]-GGF and DDHDSVDKIVIQKY concentrations were constructed and a Linear—Weighted 1/(x*x) regression applied to the data. A calibration range of 50 to 10,000 ng/mL for both analytes was determined to be the quantifiable linear range in human PBMC lysate. QC and unknown sample concentrations were determined from the appropriate calibration line and used to calculate the accuracy and precision of the method within the Study Management System, SMS2000 (Version 3.1, GlaxoSmithKline) (Table S3).

### Generation of mouse monoclonal antibody against ADM-R225-hnRNP-A1

All antibody clones were generated and purified by GenScript (NJ, USA). BALB/C mice were immunized with a synthetic peptide corresponding to amino acids 220–231 of human hnRNP-A1, GNFSG(**R**^ADMA^)GGFGGSC, according to GenScript’s proprietary rapid immunization MonoExpress protocol. Biotinylated peptides corresponding to all methylated forms of the R225-containing immunizing peptide were synthesized and purified at GenScript and used to determine the affinity and selectivity of antibodies for the asymmetrically-dimethylated form of R225-hnRNP-A1. Supernatants from the hybridoma cultures were screened against the biotinylated hnRNP-A1 peptides in a custom immunoassay based on electrochemiluminescent readout (MesoScale Discovery). Purified antibody from clone 26H3 was tested against biotinylated hnRNP-A1 peptides to determine affinity and selectivity using the custom AlphaLISA as described above.

### In vivo studies

For the PD study in blood, naïve male CD-1 mice (Charles River, MA) were dosed orally once daily with either a saline solution (vehicle) or GSK3368715 ranging from 0.137 to 600 mg/kg (n = 3 per group) for 15 days. 4 h after the last dose, mice were euthanized by carbon dioxide inhalation and full blood volume was collected in heparinized vacutainer tubes (BD, #367671) by terminal cardiac puncture. Leukocytes were purified from whole blood, incubated with red blood cell lysis buffer (Thermo Fisher Scientific, #00430054) according to the manufacturer’s specifications, and washed several times with DPBS. For the tumor xenograft model, female SCID mice (CB17.Cg-Prkdc^scid^Lyst^bg-J^/Crl, Charles River, MA) were used. A single cell suspension of Toledo cells (Human Diffuse Large B Cell Lymphoma; ATCC, CRL-2631) was prepared in 100% Matrigel (Corning, #356237) and 7 million cells delivered subcutaneously in the rear right flank of each mouse. Once tumor growth was evident, tumor volume and body weights were measured twice weekly, until the first day of dosing and then again on days 5 and 8. Tumor volumes were calculated based on the formula: tumor volume = (Length × Width^2^)/2. Following randomization into study groups (n = 4 per group) when the mean tumor size reached ~ 2000 to 2400 mm^3^, animals were dosed orally once daily with either a saline solution (vehicle) or GSK3368715 ranging from 3 to 600 mg/kg for 8 days. 24 h after the last dose, animals were euthanized by carbon dioxide inhalation and tumors were excised, butterflied, and placed in 10% buffered formalin (Fisher Scientific, #SF100-4) for 24 h. Tumor samples were then transferred to 70% ethanol and subsequently subjected to immunohistochemistry. Animals were monitored daily and any clinical observations were recorded immediately. For pharmacokinetic studies, cannulated, naive male CD-1 mice (Charles River, Raleigh, NC) or female, SCID mice CB17.B6-Prkdc^scid^Lyst^bg-J^/Crl, (Charles River, Wilmington, MA) bearing Toledo xenografts (250–400 mm^3^) were orally administered GSK3368715 at 10 mL/kg to yield a 150 mg/kg dose. CD1 mice (n = 3) were serially sampled via the femoral artery catheters at pre-dose (0), 1, 2, 4, 8 and 24 h post dose. At each time point, 25 µL blood aliquots were transferred immediately into tubes containing 25 µL distilled water, vortexed and stored frozen at − 80 °C prior to analysis. Female SCID mice bearing Toledo xenografts (n = 3/timepoint) were terminally sampled at 0.25, 0.5, 1, 2, 4, 8, 24, 48, 72, and 96 h post dosing. Blood was diluted 1:1 with water and tumors were harvested and stored at − 80 °C. Blood samples were protein precipitated using acetonitrile and analyzed by uPLC/MS/MS on a Waters Acquity uPLC (Milford, MA) and Sciex API5000 (Concord, Ontario, Canada). GSK3368715 was chromatographically separated on an Acquity BEH Phenyl, 2.1 × 50 mm column using 10 mM ammonium formate and acetonitrile using a gradient mobile phase followed by multiple reaction monitoring employing Atmospheric Pressure Chemical Ionization (APCI) in positive-ion mode. Blood pharmacokinetic parameters were obtained from the concentration–time profiles using non-compartmental analysis with Phoenix WinNonlin v6.3 (Certara, Princeton, NJ). Individual mouse profiles were used for CD1 mice while mean composite (n = 3/time point) concentration time profiles were used for SCID blood pharmacokinetic parameter determination.

All studies were conducted in accordance with the GSK Policy on the Care, Welfare and Treatment of Laboratory Animals and were reviewed by the Institutional Animal Care and Use Committee at GSK.

### Immunohistochemistry

Both total and ADM-R225-hnRNP-A1 IHC assays were developed and at QualTek Molecular Laboratories using anti-hnRNP-A1 (Abcam, #ab177152) and anti-ADM-R225-hnRNP-A1 (clone 26H3; custom development for GSK by GenScript). Human tissues were sourced from QualTek’s tissue bank and a total of 4 cases per histology were evaluated. Human tissues and tumor xenografts were freshly cut at a thickness of 4–5 μm and mounted onto positively-charged, capillary gap glass slides prior to use. Briefly, slides were baked at 60 °C (dry heat) and subjected to antigen/epitope retrieval using a steam heat induced epitope recovery (SHIER) solution for 20 min in the capillary slide gap in the upper chamber of a Black and Decker Steamer, as previously described by Ladner et al*.*^31^. The SHIER solutions used for antigen retrieval of total hnRNP-A1 and ADM-R225-hnRNP-A1 were AR10 (pH 10) and citrate-based (pH 6.0–6.2), respectively. Following antigen retrieval, all process steps were automated using a TechMate Instrument (Roche Diagnostics) running QML workmate software v3.96. After blocking in 5% goat serum for 15 min, slides were incubated at room temperature either with the hnRNP-A1 antibody over night or with the ADM-R225-hnRNP-A1 antibody for 1 h. Slides were then washed in tris-buffered saline with 0.15% Tween-20 (TBS-T) and incubated with either mouse or rabbit Polink-2 Plus secondary antibodies (GBI Labs) for 25 min at room temperature. Following washes in TBS-T, slides were blocked in hydrogen peroxide (3 × 2.5-min incubations) and incubated with Polink-2 Plus Horseradish Peroxidase (HRP, GBI Labs) for 25 min at room temperature. Slides were then exposed to a chromogen substrate (DAB) (3 × 2.5-min incubations) and counterstained with hematoxylin. Slides were rinsed in distilled water, dehydrated in both an alcohol series (95%, 100% ethanol) and in an organic solvent (100% xylene, four changes), then permanently coverslipped using CytoSeal 60 (Thermo Fisher scientific) and reviewed on an Olympus BX60F5 microscope using 20-40X objectives. Mouse IgG1 and rabbit IgG isotype-match controls corresponding to the IHC assay conditions for total hnRNP-A1 and ADMA-R225 were included on each tissue tested. All tissues analyzed were scored by a board-certified pathologist and the levels of hnRNP-A1 protein, both total and ADM-R225, are reported as H-Scores, and calculated by summing the percentage of cells with intensity of expression (brown staining) multiplied by their corresponding differential intensity on a four-point semi-quantitative scale (0, 1 + , 2 + , 3 +). H-Scores range from 0 to 300 based on the following equation:

$${\text{H-Score}} = \left[ {\left( {\% {\text{ at }} < {1}} \right) \times 0} \right] \, + \, \left[ { \, \left( {\% {\text{ at 1}} + } \right) \times {1}} \right] \, + \, \left[ { \, \left( {\% {\text{ at 2}} + } \right) \times {2}} \right] \, + \, \left[ { \, \left( {\% {\text{ at 3}} + } \right) \times {3}} \right]$$

Representative regions of interest were identified visually, and high-resolution images were taken from a live microscope view using Amscope software.

### Purification of immune cells from human blood

Human immune cells from healthy donors were obtained through GSK’s Blood Donation Unit. All donors provided written informed consent prior to blood collection. The use of these samples was reviewed and approved by GSK Research & Development Compliance (RDC) Human Biological Sample Use Committee. White blood cells were purified from whole blood collected in heparin- (APP pharmaceuticals, #NDC63323-540-11) containing syringes by incubation with red blood cell lysis buffer (Thermo Fisher Scientific, #00430054) following the manufacturer’s instructions, and washed several times with DPBS. PBMCs were isolated from whole blood collected in Vacutainer Mononuclear Cell Preparation Tubes (CPT) containing sodium citrate (BD, #362761) according to the manufacturer’s instructions. Monocytes were purified from fresh PBMCs, obtained via Ficoll-Paque according to the manufacturer’s instructions^32^ (Sigma-Aldrich, #GE17-1440-02), using the Pan Monocyte Isolation Kit (Miltenyi, #130096537), as per the manufacturer’s protocol. Neutrophils were obtained from whole blood using the MACSxpress Whole Blood Neutrophil Isolation Kit (Miltenyi, #130104434) according to the manufacturer’s instructions. For whole-cell lysis, 10–30 million of purified cells were washed with DPBS (Thermo Fisher Scientific, #14190144), lysed with 20 µL RIPA buffer (Sigma-Aldrich, #R0278) + 1X Protease Inhibitor Cocktail (Sigma-Aldrich, P8340) per million cells, and incubated on ice for 10 min. Homogenized samples were applied to a QIAshredder column (Qiagen, #79656), and centrifuged at 12,000 × RCF for 2 min. Protein extracts were quantified using the BCA Protein Assay Kit (Pierce, #23225).

### Flow cytometry

Flow cytometric analysis of the isolated cell populations was performed as follows: 2 × 10^5^ cells were stained with Live/Dead Fixable Near IR dead cell stain kit (prepared as per manufacturer’s instructions; ThermoFisher Scientific; #L34975) for 30 min, in the dark, at 4 °C. After washing, cells were incubated with human FcR Blocking Reagent (Miltenyi Biotec; #130-059-901), for 15 min, in the dark, at 4 °C, to block nonspecific binding of antibodies. Cells were then incubated for 30 min, in the dark at 4 °C, in Brilliant Stain buffer (BD Pharmingen; #563794) with antibodies against the indicated surface proteins (as per manufacturer’s instructions): BV510 Mouse Anti-Human CD45 (BD Biosciences, #563204), FITC Mouse Anti-Human CD3 (BD Biosciences, #561807), PerCP-Cy5.5 Mouse Anti-Human CD16 (BD Biosciences, #560717), PE Mouse Anti-Human CD14 (BD Biosciences, #557154), PE-Cy7 Mouse Anti-Human CD20 (BD Biosciences, #560735), and Alexa Fluor 700 anti-human CD15 (BioLegend, #301920), or isotype controls: BV510 Mouse IgG1, κ (BD Biosciences, #562946), FITC Mouse IgG1, κ (BD Biosciences, #555748), PerCP-Cy5.5 Mouse IgG1, κ (BD Biosciences, #550795), PE Mouse IgG2a, κ (BD Biosciences, #556653), PE-Cy7 Mouse IgG2b, κ (BD Biosciences, #560542), and Alexa Fluor 700 Mouse IgG1, κ (BioLegend, #400144). Cells were then washed and fixed with Fixation Buffer (BD Biosciences, #554655) for 10–15 min, in the dark, at 4 °C. After washing, cells were resuspended with Stain buffer (BD Biosciences, #554657) and acquired on a BD FACSCanto 10 flow cytometer after setting up the proper compensation. The data were analyzed by FlowJo (v10.1r5; Treestar) software.

### Quantification and statistical analysis

Graphing of results and statistical analyses were performed using Microsoft Excel. Sample sizes are indicated in the figure legends and data are expressed as the mean ± standard deviation (SD). Where applicable, statistical significance was evaluated using either a two-tailed Student’s *t*-test (Microsoft Excel) or the Mann–Whitney test (Study Director software). IC_50_s were calculated in Xlfit (IDBS) using a 4 parameter logistic model (sigmoidal dose–response). Figure 1 was created using ChemDraw (PerkinElmer) and Microsoft PowerPoint.

### Use of human biological samples

For all studies involving human biological samples, methods were performed in accordance with the relevant guidelines and regulations, and the use of these samples was reviewed and approved by GSK Research & Development Compliance (RDC) Human Biological Sample Use Committee. Written informed consents and/or a waiver of consent approved by an Institutional Review Board (IRB) in accordance with all applicable laws were provided for all study participants.

## Supplementary Information

Supplementary Data File 1.

Supplementary Data File 2.

Supplementary Materials.

Supplementary Figure S7.
