# Supplementary material for: Identification of hnRNP-A1 as a pharmacodynamic biomarker of type I PRMT inhibition in blood and tumor tissues
Source: Sci Rep. 2020 Dec 17;10:22155. doi: 10.1038/s41598-020-78800-6 (PMC7746746; doi:10.1038/s41598-020-78800-6)

# **Title**

**Identification of hnRNP-A1 as a pharmacodynamic biomarker of type I PRMT inhibition in blood and tumor tissues.**

# **Authors:** Paul B. Noto ^1*^, Timothy W. Sikorski^2^, Francesca Zappacosta^3^, Craig D. Wagner^3^, Rocio Montes de Oca^1^, Matthew E. Szapacs^2^, Roland S. Annan^3^, Yan Liu^4^, Charles F. McHugh^5^, Helai P. Mohammad^4^, Steven P. Piccoli^1,6^ & Caretha L. Creasy^4^

# **Affiliations**:

^1^ Experimental Medicine Unit, Oncology R&D, GSK.
^2^ Protein Mass Spectrometry, In Vitro / In Vivo Translation, Research, GSK.
^3^ Discovery Analytical, Medicinal Science and Technology, Research, GSK.
^4^ Epigenetics, Oncology R&D, GSK.
^5^ Discovery DMPK, In Vitro / In Vivo Translation, Research, GSK
^6^ Clinical Biomarkers and Diagnostics, Sun Pharmaceutical Advanced Research Center (SPARC).

^*^Corresponding author: Paul B. Noto, paul.x.noto@gsk.com.

# **Supplementary Materials:**

## **Figure S1**


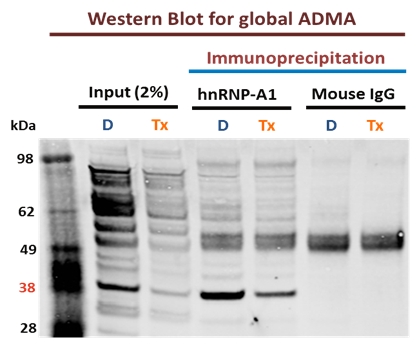


**Figure S1. Immunoprecipitation of hnRNP-A1 from Toledo cells.** Western blot for ADMA in lysates (input) and immunoprecipitated hnRNP-A1 from cells treated with GSK3368712B for 48 hours**.** D, DMSO. Tx, treatment with 1 μM GSK3368712B.

## **Figure S2**

**Figure S2. LC-MS/MS analysis of R225 on hnRNP-A1 in human PBMCs.** Analysis of R225 methylation status by monitoring of monomethylation (MMA) and dimethylation (DMA), in the form of either ADMA or SDMA, in both non-stimulated and TCR-activated human PBMCs treated with DMSO for 72 hours.

## **Figure S3**

**Figure S3. LC-MS/MS detection of SDMA and ADMA forms of R225-hnRNP-A1 peptides.** (**A**) Resolution of synthetic hnRNP-A1 peptides containing R225 that is either symmetrically or asymmetrically-dimethylated. (**B**) Co-detection of endogenous ADM-R225-hnRNP-A1 peptide in non-stimulated, non-treated, human PBMCs and synthetic ADM-R225-hnRNP-A1 internal standard peptide.

## **Figure S4**


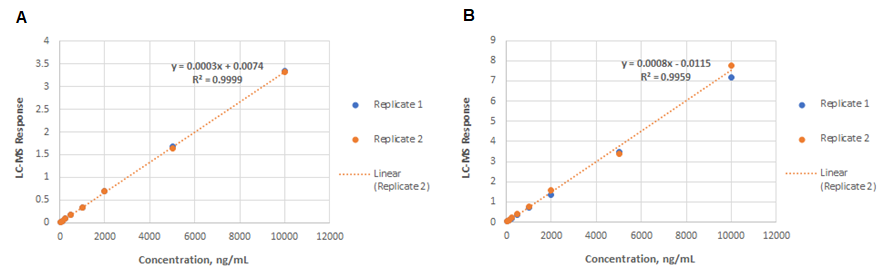


**Figure S4. LC-MS/MS detection of peptides from chymotrypsin-digested recombinant hnRNP-A1 protein.** Linear regression of (**A**) DM-R225-hnRNP-A1 (aa. 223-228) and (**B**) hnRNP-A1(aa. 154-167). LLOQ for both peptides determined to be 50 ng/mL.

## **Figure S5**

**Figure S5. Flow cytometry analysis of leukocytes from human blood.** (**A**) whole blood after red blood cell (RBC) lysis. (**B**) B and T cells populations, (**C**) monocytes and (**D**) neutrophils from RBC-lysed whole blood (CD45^+^). (**E**) PBMCs and B and T cells (**F**) obtained from CPT tubes. (**G**) Monocytes purified from freshly isolated PBMCs. (**H**) Neutrophils purified directly from whole blood.

## **Figure S6**

**Figure S6. Western Blot analysis of hnRNP-A1.** Detection of ADM-R225-hnRNP-A1, using the novel antibody (clone 26H3), and total hnRNP-A1 in Toledo cells and non-stimulated human PBMCs treated with either DMSO or 2μM GSK3368712 for 48 and 72 hours, respectively; recombinant hnRNP-A1 protein expressed in prokaryotic (E.coli) and eukaryotes (human kidney cells, HEK293) were included as controls.

## **Figure S7**

***See Figure S7 on the editorial system***

**Figure S7. IHC staining with species-specific isotype controls.** IHC staining for mouse and rabbit IgG antibodies across a panel of tumor tissues and tonsil.

## **Figure S8**


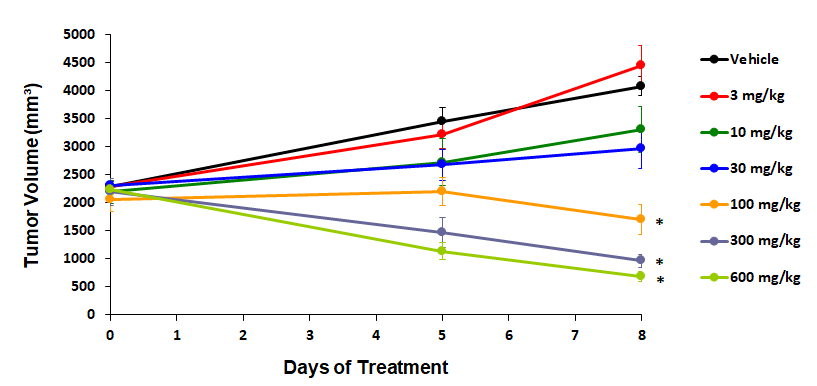


**Figure S8. Inhibition of tumor growth in a Toledo xenograft model.** Mice engrafted with a human Toledo cancer line were treated with oral, once-daily doses of GSK3368715 for 7 days. Tumor volumes were measured on days 5 and 8, prior to collection of tumors on day 8. *, *p* =0.0304 (Mann-Whitney test) from comparison of GSK3368715-treated Vs. vehicle-treated animals.

## **Table S1.** **Accuracy, Precision and Individual Quality Control Sample Concentrations for DM-RNPA1 and RNPA1_NORM in Human PBMC Lysate**


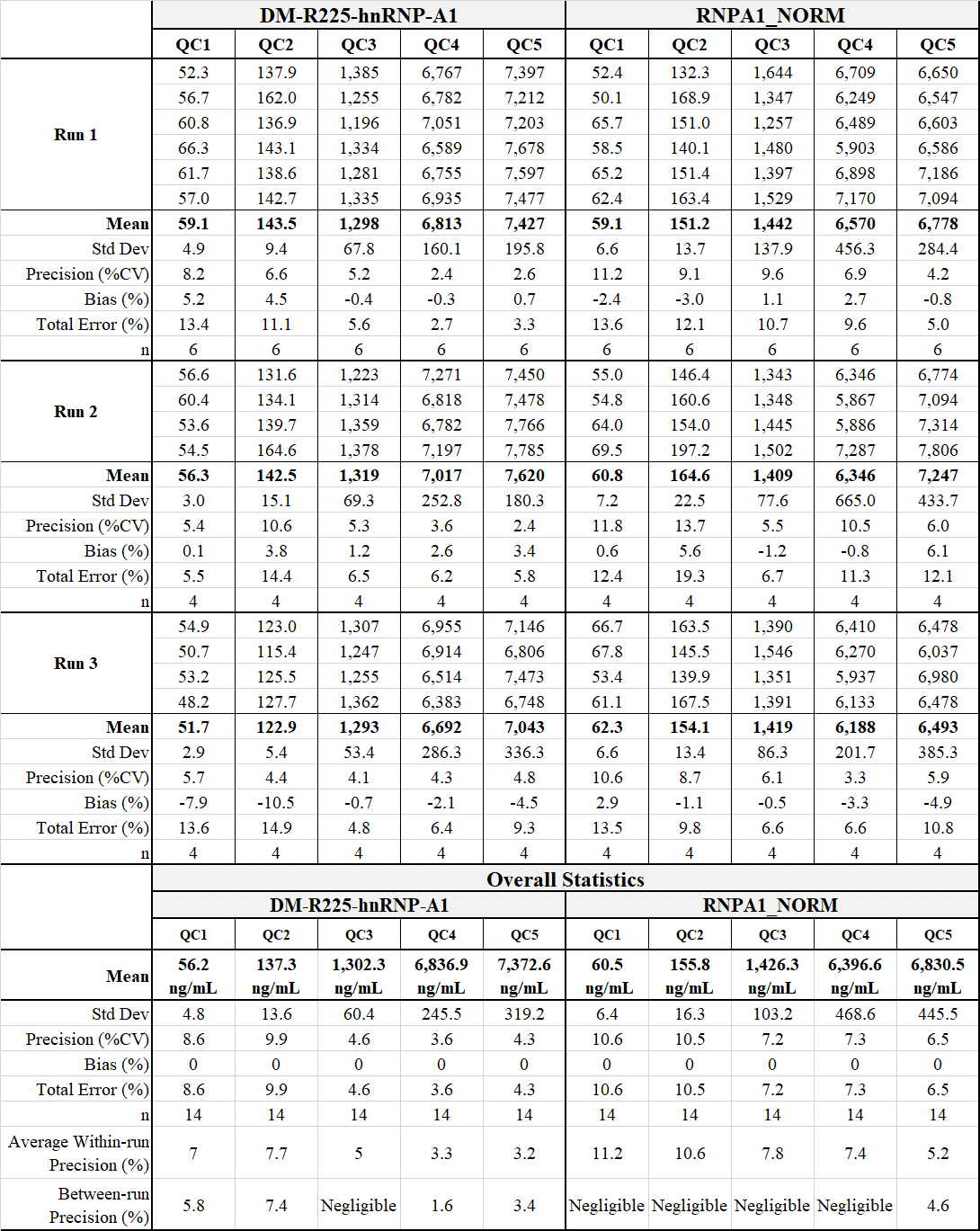


## **Table S2. Assessment of pre-analytical variables in the quantitation of hnRNP-A1 peptides in human PBMCs by LC-MS. (A)** Comparison of PBMC pellets processed and analyzed immediately upon isolation (fresh) or after storage at -70ºC for 13 weeks (**B**) Stability of hnRNP-A1 peptides assessed in PBMC lysates after 3 freeze-thaw cycles or storage at -70ºC for 8 months.

| **A** | **SAMPLE** | **Donor-Pool** | **Generation of Lysate & Analysis** | **DMA-RNPA1 (ng/mL)** | **% Change** | **NORM-RNPA1 (ng/mL)** | **% Change** |
| --- | --- | --- | --- | --- | --- | --- | --- |
|  | PBMC pellet | **1** | Immediately upon PBMC isolation | 803.2 | baseline | 862.8 | baseline |
|  |  |  | After 13 week-storage at -70°C | 720.7 | -10.30% | 748.6 | -13.20% |
| **B** | PBMC Lysate | **2** | After single freeze-thaw cycle | 1,302.3 | baseline | 1,426.3 | baseline |
|  |  |  | After 3 additional freeze-thaw cycles | 1,212.8 | -6.90% | 1,624.2 | 13.90% |
|  |  |  | After storage of lysate for 8 months at -70°C | 1,374.6 | 5.50% | 1,513.7 | 6.20% |

## **Table S3. Affinity of ADM-R225-hnRNP-A1 antibody clones for non-and methylated forms of an hnRNP-A1 peptide.** Selectivity calculated as the ratio between the electrochemiluminescent (ECL) units measured for the asymmetrically-dimethylated peptide over the units for unmethylated, mono-methylated and symmetrically-dimethylated hnRNP-A1 peptides.

## **Table S4. H-scores for ADM-R225-hnRNP-A1 and total hnRNP-A1 in tumor tissues obtained from mice engrafted with Toledo cells and treated with GSK3368715 orally once-daily for 8 days (n=3).**

## **Table S5. Blood levels of GSK3368715 in immunocompetent (CD1) and SCID mice after a single dose of 150 mg/kg.**

## **Table S6. MS/MS Transition List**


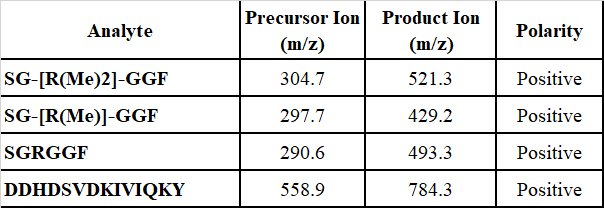

Supplement: Supplementary file 3 — Supplementary Materials. [file 41598_2020_78800_MOESM3_ESM.docx]
