# Supplementary material for: Identification of hnRNP-A1 as a pharmacodynamic biomarker of type I PRMT inhibition in blood and tumor tissues
Source: Sci Rep. 2020 Dec 17;10:22155. doi: 10.1038/s41598-020-78800-6 (PMC7746746; doi:10.1038/s41598-020-78800-6)
Supplement: Supplementary file 4 — Supplementary Figure S7. [file 41598_2020_78800_MOESM4_ESM.docx]

## **Figure S7**
